# Report on a Case of Reunited Fractured Human Tooth

**Published:** 1896-11

**Authors:** Storer Bennett


### Abstracts and Translations.

### REPORT ON A CASE OF REUNITED FRACTURED HUMAN TOOTH.

BY MR. STORER BENNETT.

In December, 1888, Mr. W. E. Harding presented to this Society an upper incisor which had been fractured across the crown, and which he had extracted from the mouth of a girl, seventeen years of age, only three or four days before he presented it. The history of the patient was that some ten months previously she had fallen down, striking the tooth and driving it high up in its socket. It became impacted, and remained fixed in its position, causing more and more irritation up to the time that Mr. Harding saw it. The pain gradually became so intense that there was nothing to do but to remove the tooth. He then discovered that it had been fractured across the crown, in a direction obliquely upward and backward. I was asked to make a microscopic examination of the specimen, and, therefore, removed a vertical section from front to back of the tooth, and the slide now exhibited shows the two outer halves remaining. It was seen that the broken portions of the specimen, though separated by a considerable interval, were firmly knit together by some calcified material which occupied the central portion of the gap. The margins of the space, however, being occupied by a substance of leathery consistence, were not calcified at all. Examined microscopically, the uniting substance was seen to consist of a calcified material of a spongy or cavernous character, with numerous spaces for blood-vessels. The cavernous spaces had apparently been occupied by a substance somewhat resembling pulp, though I do not wish to affirm that it was pulp. In various positions slight absorption of the edges of the normal dentine has taken place, the spaces thus formed being filled up by cementum showing well-marked lacunæ and canaliculi. The amount of cementum, however, is not very great.

The next slide is a magnification showing the intermediate tissue. Spaces are seen which have been occupied by blood-vessels, and from the black masses little tubes here and there may be seen passing, but there is no space where one can make out a distinct brush of tubes going off, as one would expect if a mass of dentine were present.

The next slide shows a smaller portion of the same thing more highly magnified, but I do not know that it adds any greater amount of clearness to the idea of the specimen. An examination of the specimen suggests to one's mind two different sources for the supply of this new material, either pulp or periosteum. The pulp was exposed, but only to a very slight extent, and, of course it is possible to imagine that enlargement took place, that the overgrowth of the pulp filled up the space between the two fragments somewhat similarly to the way that chronic enlargement occurs in cases of polypus of the pulp, and that ultimately this calcified.

On the other hand, we have evidence that there is cementum in the section, lacunæ and canaliculi being present in certain parts in rather large numbers, and we have *not* the evidence of any definite dentine structure asserting itself. We know that bone may be produced from many tissues other than those which naturally give rise to it. We have here, I think, a case in which hemorrhage having taken place, a natural capping of the exposed pulp occurred, somewhat similarly to the way a wound heals under a scab. Blood was poured out between the fragments, organization took place, numerous blood-vessels were produced, and ultimately calcification occurred, and eventually, if it had been left long enough, I think the whole of the space would have been filled up with calcified material more or less resembling bone, or bone and cementum together. There are many specimens described where cementum has united a fractured tooth in the root, but I think we have here to do with a specimen unlike any other that has been figured or described, in so far as the cementum has been deposited between the fractured portions of the crown.

